# Effects of CORO2A on Cell Migration and Proliferation and Its Potential Regulatory Network in Breast Cancer

**DOI:** 10.3389/fonc.2020.00916

**Published:** 2020-06-26

**Authors:** Jun-Li Deng, Hai-Bo Zhang, Ying Zeng, Yun-Hua Xu, Ying Huang, Guo Wang

**Affiliations:** ^1^Department of Clinical Pharmacology, Xiangya Hospital, Central South University, Changsha, China; ^2^Hunan Key Laboratory of Pharmacogenetics, Institute of Clinical Pharmacology, Central South University, Changsha, China; ^3^Engineering Research Center of Applied Technology of Pharmacogenomics, Ministry of Education, Changsha, China; ^4^National Clinical Research Center for Geriatric Disorders, Central South University, Changsha, China

**Keywords:** CORO2A, breast cancer, migration, proliferation, prognosis, regulatory network

## Abstract

Coronin 2A (CORO2A) is a novel component of the N-CoR (nuclear receptor co-repressor) complex. Abnormal CORO2A expression is associated with carcinogenesis. We used databases from the Cancer Genome Atlas (TCGA) and Gene Expression Omnibus (GEO), and analyzed CORO2A expression and gene regulation networks in breast cancer. Expression was analyzed using GEO and TCGA database and further validated in breast cancer samples collected in our clinic. The prognostic value of CORO2A was explored by using the Kaplan–Meier survival analysis and Cox proportional hazards regression analysis. LinkedOmics was used to identify coexpressed genes associated with CORO2A. After analyzing the intersection of coexpressed genes correlated with CORO2A and differentially expressed genes after CORO2A silencing, Gene Ontology (GO) and Kyoto Encyclopedia of Genes and Genomes (KEGG) pathway analyses of the intersecting genes were conducted by using *FunRich* software. Transwell assays were performed in breast cancer cells to determine the effect of CORO2A on cell migration. MTS, colony formation, and cell cycle distribution assays were performed in breast cancer cells to determine the effect of CORO2A on cell proliferation. Gene enrichment analysis was employed to explore the target networks of transcription factors and miRNAs. We found that CORO2A was upregulated and that the elevated expression of CORO2A was associated with poor overall survival (OS) and relapse-free survival (RFS) in TNBC patients. Further bioinformatics analysis of public sequencing data and our own RNA-Seq data revealed that CORO2A was probably involved in the epithelial-to-mesenchymal transition process and might have a significant effect on the migration of breast cancer cells, which might be mediated via pathways involving several miRNAs and MYC transcription factors. Functionally, the knockdown of CORO2A inhibited cell migration, decreased viability, and colony formation and induced cell cycle arrest in the G0/G1 phase in breast cancer cells. These results demonstrate that bioinformatics-based analysis efficiently reveals information about CORO2A expression and its potential regulatory networks in breast cancer, laying a foundation for further mechanistic research on the role of CORO2A in carcinogenesis. Moreover, CORO2A promotes the migration and proliferation of breast cancer cells and may have an important function in breast cancer progression. CORO2A is a potential prognostic predictor for TNBC patients. Targeting CORO2A may provide promising therapy strategies for breast cancer treatment.

## Introduction

Breast cancer is reported to be the most common malignancy in women, with an estimated 268,600 new cases and 41,760 deaths in 2019, which represents 30% of all new cancer cases and 15% of cancer-related deaths among American women (1, 2). Breast cancer is a complex disease with cumulative genetic and epigenetic aberrations as well as cancer cell heterogeneity (3). Understanding these pathological molecular alterations has significant implications in cancer initiation and progression as well as in cancer prevention and treatment. Therefore, the identification of the potential key molecules involved in the development and progression of breast cancer, which may function as biomarkers for the diagnosis and prognosis of breast cancer, is urgently needed.

In our previous study, by using four public datasets from the Gene Expression Omnibus (GEO) and TCGA, we found that CORO2A was overexpressed in malignant breast tissues compared to normal breast tissues (4). Coronin 2A (CORO2A), also known as CLIPINB, IR10, or WDR2, is a member of the “short” coronin subfamily containing a single WD40-repeat domain, which adopts the fold of a seven-bladed β-propeller (5). Compared to other well-characterized coronins, less information is available about CORO2A. In 2003, Yoon et al. (6) revealed that CORO2A is a novel component of the N-CoR (nuclear receptor co-repressor) complex. In addition, Huang et al. (7) uncovered that CORO2A mediates actin-dependent derepression of inflammatory response genes, which is regulated by phosphorylation and by nuclear receptor signaling pathways. Rastetter et al. (8) illuminated that the expression level of CORO2A is related to the malignant progression of colon carcinoma and that the overexpression of CORO2A increases tumor cell migration. Recently, Fridley et al. (9) observed shorter progression-free survival (PFS) in endometrioid carcinoma and clear cell carcinoma patients with high gene expression for CORO2A. Based on these studies, we studied the function and prognostic significance of CORO2A in breast cancer.

In the current study, we found that patients with elevated expression of CORO2A had poorer overall survival (OS) and relapse-free survival (RFS) in TNBC via Kaplan–Meier survival analysis. Loss-of-function experiments further indicated that knockdown of CORO2A reduces cell migration, cell viability, and colony-formation and induces cell cycle arrest in the G0/G1 phase of breast cancer cells. In addition, we also studied CORO2A expression in data from patients with breast cancer in The Cancer Genome Atlas (TCGA) and various public databases. Using multidimensional analysis methods, we analyzed the functional networks and validated the function of CORO2A in breast cancer. These findings suggest that CORO2A is a novel oncogene that may be involved in breast cancer initiation and progression, and functions as a potential biomarker for the diagnosis and prognosis of breast cancer.

## Materials and Methods

### Expression Analysis of CORO2A in UALCAN

UALCAN (http://ualcan.path.uab.edu.) is a user-friendly, interactive web resource for analyzing cancer transcriptome data (TCGA and MET500 transcriptome sequencing) from 31 cancer types (10). UALCAN allows users to identify biomarkers or to perform *in silico* validation of potential genes of interest. One of the portal's user-friendly features is that it allows analysis of relative expression of a query gene(s) across tumor and normal samples, as well as in various tumor molecular subtypes such as individual age of diagnosis, gender, stages, or other clinicopathological features, which allow us to explore the relative expression of CORO2A in breast cancer.

### Bioinformatic Data Mining of TCGA Database

The breast cancer expression profile data and corresponding clinical data were downloaded from the TCGA database (http://tcga-data.nci.nih.gov). And the clinical characteristics of the patients were shown in Table 1.

**Table 1:** Clinical characteristics of breast cancer (BC) patients.

**Clinical characteristic**	**Breast cancer**	**TNBC**	**Luminal BC**	**Undefined BC**
No. of patients	1,087	135	681	271
**Age (years)**				
≥60	502	41	321	140
<60	585	94	360	131
**Pathologic stage**				
I	182	23	115	44
II	617	90	375	152
III	246	17	161	68
IV	20	2	12	6
Undefined	22	3	18	1
**Lymphnodes**				
Positive	475	43	303	129
Negative	456	82	261	113
Undefined	156	10	117	29

“*Undefined BC” means that provided information on estrogen receptor (ER), progesterone receptor (PR), and HER2 expression are deficient, so those patients cannot be classified as any subtype of breast cancer*.

### LinkedOmics Analysis

LinkedOmics is a publicly available platform for analyzing multiomics data from all 32 TCGA cancer types and mass spectrometry-based proteomics data, especially for TCGA breast, colorectal, and ovarian tumors (11). LinkedOmics is publicly available at http://www.linkedomics.org/login.php. Since the *LinkFinder* module of LinkedOmics allows users to search for attributes that are associated with a query attribute, such as mRNA, we investigated genes that were differentially expressed in correlation with CORO2A in the TCGA breast cancer cohort. The results were analyzed by Pearson's correlation coefficient and visualized by heat maps, volcano plots, or scatter plots. In addition, data from the *LinkFinder* module were ranked, and GSEA was adopted to conduct analyses of transcription factor-target enrichment and miRNA-target enrichment. The two network analyses were based on the Molecular Signatures Database (MSigDB) (12). The rank criterion was set as FDR < 0.05, and 1,000 simulations were performed.

### RNA-Seq Analysis

BT549 cells were plated in six-well-plates. After 24 h, the cells were then transfected with CORO2A siRNA using RNAiMAX reagent (Invitrogen, Carlsbad, CA, USA). Total cellular RNA was extracted and quantified by a NanoDrop ND-2000 (Thermo Scientific, Massachusetts, USA). The RNA integrity was assessed using an Agilent Bioanalyzer 2100 (Agilent Technologies, California Palo Alto, USA). RNA-Seq was performed and analyzed by Novogene (Beijing, China) under the HiSeq 4000 Illumina platform. The threshold set for up- and down-regulated genes was a fold change ≥ 2.0 and a *P* < 0.05. Finally, hierarchical clustering was performed to display the distinguishable gene expression pattern among samples.

### Enrichment Analysis of Intersecting Genes

The intersecting genes of genes coexpressed with CORO2A and differentially expressed genes after CORO2A silencing were analyzed in human breast cancer. Briefly, first, the intersecting genes from downregulated genes after CORO2A silencing from RNA-seq analysis and genes positively correlated with CORO2A expression from LinkedOmics analysis were obtained. Second, the intersecting genes from upregulated genes after CORO2A silencing from RNA-seq analysis and genes negatively correlated with CORO2A expression from LinkedOmics analysis were also obtained. Next, *FunRich* software (*FunRich_V3*) was applied to perform Gene Ontology (BP and MF) and biological pathway enrichment analysis of these intersecting genes.

### GeneMANIA Analysis

GeneMANIA (http://www.genemania.org) is a flexible, user-friendly web tool for the construction of a protein-protein interaction (PPI) network, for generating hypotheses about gene function, analyzing gene lists and prioritizing genes for functional assays (13). Therefore, GeneMANIA was used to visualize the gene networks of genes that were identified as being enriched in breast cancer: transcription factor MYC (Figure 10) and miRNA-493 (Supplementary Figure 2).

### Cell Culture

The MDA-MB-231 and BT549 human breast cancer cell lines were obtained from the American Type Culture Collection (ATCC) (Rockefeller, Maryland, USA). Both breast cancer cell lines were cultured in RPMI-1640 medium (BI, Kibbutz Beit HaEmek, Israel) supplemented with 10% fetal bovine serum (FBS, BI), 2 mM glutamine (BI), 100 U/ml penicillin (BI), and 100 μg/ml streptomycin (BI) at 37°C in a 5% CO2 atmosphere.

### Transfection

SiRNA knockdown of CORO2A was performed as follows. siCORO2A-1, siCORO2A-2, and negative control siRNA (NC) were obtained from Genepharma (Genepharma, Shanghai, China). Briefly, MDA-MB-231 and BT549 cells were plated in six-well-plates. After 24 h, the cells were then transfected with 50 nM siRNA using RNAiMAX reagent (Invitrogen, Carlsbad, CA, USA) according to the manufacturer's instructions. The sequences of the siRNAs were as follows: si-CORO2A-1 GACCTATCTTCAATTCCAT; and si-CORO2A-2 GCGAGATCTTCCGCTTCTA.

### Western Blotting

Western blot analysis was conducted as previously described (14). Briefly, protein extracts from harvested experimental cells were prepared using lysis buffer from Beyotime Biotechnology (Shanghai, China). Thirty micrograms of protein extracts were loaded onto a 12% SDS-PAGE gel, electrophoretically separated, and transferred to polyvinylidene membranes (Beyotime Biotechnology, Shanghai, China). After incubating the membrane with 5% non-fat milk blocking buffer at room temperature for 1 h, the membranes were incubated with primary antibody overnight at 4°C. After incubation with a horseradish peroxidase-conjugated secondary antibody (1:2,500, Promega, Madison, WI, USA) at room temperature for 1 h, the signal was obtained by using an ECL Western blotting system (Promega, Madison, WI, USA) and visualized and quantified using the Bio-Rad ChemiDoc MP system. The primary antibodies against CORO2A (Sigma, catalog # HPA041302, 1:1,000) were obtained from Sigma (Sigma Chemical Co., St. Louis, MO, USA). β-actin, also obtained from Sigma, was used as the loading control.

### Immunostaining

Immunofluorescence experiments were performed according to the instructions of the Fast ImmunoFluorescence Staining Kit (Protein Biotechnologies). The primary antibody was anti-CORO2A (Sigma, catalog # HPA041302, 1:400). The secondary antibody was Alexa Fluor 488 (Jackson ImmunoResearch). The nucleus was detected using DAPI Fluoromount-G® (Beyotime Biotechnology). The images were collected by using DeltaVision Elite Imaging System (GE Healthcare Life Sciences).

### Scratch-Wound Assay

After transfection with CORO2A plasmids for 24 h, cells were seeded into a 12-well-culture plate until a monolayer of cells was formed, and then a straight line was scratched on the monolayer of cells with a sterile 1,000-μl pipette tip. Then, the culture medium was removed, and the wells were washed thrice with the culture medium to remove the debris. The width of the scratch area was measured 24 h later to estimate the migration capacity.

### Transwell Assay

Cellular migration was evaluated using Transwell migration chambers precoated with a layer of Matrigel® (Corning Cat# 3422). Cells suspended in 100 μl medium without FBS were seeded in the upper chamber (3 × 10^4^ cells/well) following transfection with CORO2A siRNA or control for 24 h. To the lower chamber, 600 μl medium supplemented with 10% FBS was added. After 24 h incubation at 37°C, cells on the top of the membrane were removed with cotton swabs. The cells that migrated through the membrane were washed with PBS, fixed in 4% methanol for 20 min and stained with 0.1% crystal violet for 10 min at room temperature. The number of migratory cells was counted in five randomly selected fields under a light microscope (Leica Microsystems GmbH, Wetzlar, Germany).

### Cell Viability Assay

The MTS assay was performed to detect viable cell numbers using a cell proliferation assay kit from Promega (Madison, WI, USA). For the MTS assay, MDA-MB-231 and BT549 cells were seeded in 96-well-plates at 2 × 10^3^ cells/well, cultured for 24 h in growth media, and then treated with CORO2A siRNA for 24, 48, 72, and 96 h. The absorbance (A) at 490 nm was detected using a microplate reader.

### Cell Colony Formation Assay

The cell colony formation assay was conducted as previously described with minor modifications (15). Briefly, 1 × 10^3^ cells/well transfected with CORO2A siRNA or control for 24 h were initially seeded in a six-well-plate. The medium was refreshed every 2 days. At day 10 of treatment, when the colonies were visible by the naked eye, the cells were fixed with 4% paraformaldehyde (Sigma-Aldrich) for 30 min, stained with 1% crystal violet for 20 min and then counted. The colony numbers were quantified using *ImageJ* software.

### Cell Cycle Assay

Briefly, after transfection with CORO2A siRNA for 48 h, 1 × 10^6^ cells were collected, trypsinized, and fixed in 70% ethanol overnight. Then, the cells were washed three times with precooled PBS and incubated with a PI-staining solution with RNase A (Beyotime Biotechnology, Shanghai, China) for at least 15 min at room temperature before analysis. The cells were then run on a FACScan cytometer (BD Biosciences, America) in accordance with the manufacturer's guidelines.

### Patients and Tissue Samples

All 22 breast cancer tissues and paired adjacent non-cancerous tissues were obtained from patients who had undergone radical surgical resection of breast cancer from March 2015 to December 2016 at Xiangya Hospital of Central South University, China. The paired adjacent non-cancerous tissues were dissected by the surgeons 5 cm away from the tumor edge. Tissue samples were stored in liquid nitrogen until total RNA was extracted. These breast cancer patients were diagnosed and graded by pathological features in the Department of Pathology, Xiangya Hospital. This study was approved by the Ethics Committee of Xiangya Hospital, and informed consent forms (IFCs) were obtained from all patients. The clinicopathological characteristics of the 22 breast cancer patients are shown in Supplementary Table 1.

### Quantitative Real-Time RT-PCR

Briefly, 1 μg of total RNA was reverse transcribed in a 20 μl reaction using the PrimeScript™ RT reagent kit with gDNA Eraser (Takara, Dalian, China, Code No: RR047A) according to the manufacturer's protocol. The reaction products were then diluted with 80 μl distilled water. The real-time PCR was composed of 2 μl of diluted reverse transcription product, 10 μl of 2× SYBR® Premix DimerEraser™ (Takara Bio Inc., Code No: RR091A) and 0.6 μl of forward and reverse primers (0.3 μM). The reaction was performed in a Light Cycle^@^ 480 II Sequence Detection System (Roche, Basel, Switzerland) for 40 cycles (95°C for 5 s, 55°C for 30 s, 72°C for 30 s) after an initial 30 s denaturation at 95°C. β-actin was used as an internal control. The RNA levels of tumor samples and paired adjacent samples were calculated using the 2^−Δ*Ct*^ method. The primers for CORO2A and β-actin were synthesized by Sangon Bio-tech (Shanghai, China), and their sequences are listed in Supplementary Table 2.

### Statistical Analysis

Each experiment was performed at least three times, and data are expressed as the mean ± SEM. Statistical analyses were performed using SPSS 23.0 software (SPSS, Inc., Chicago, IL, USA). One-way ANOVA was applied to determine the difference among multiple groups, and Student's *t*-test was adopted to compare the statistical significance between two groups. Kaplan–Meier method and log-rank test were applied to compare the survival rate. Multivariate Cox proportional hazard models were used to analyze the effect of clinical variables on patients' OS and RFS. A *p* < 0.05 was considered to be statistically significant.

## Results

### CORO2A Expression in Breast Cancer

**Figure 1:**
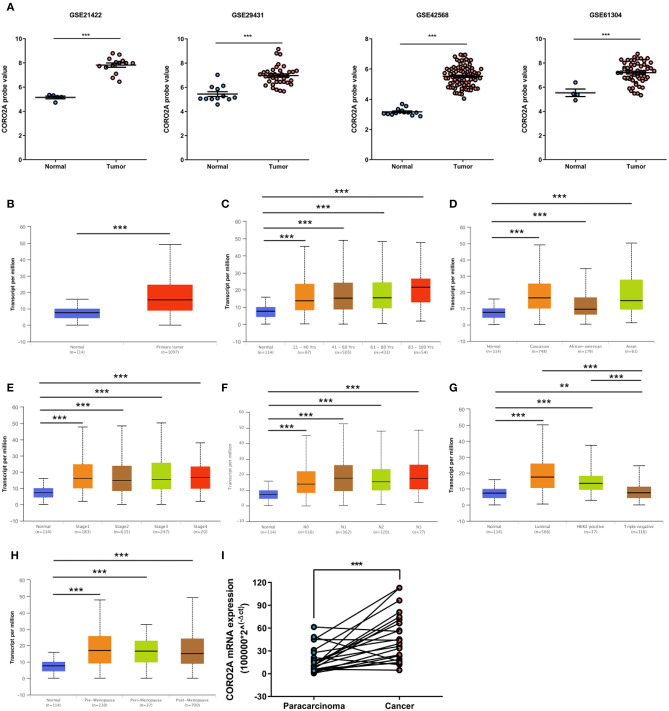
Relative expression of CORO2A in malignant and non-malignant breast tissues. **(A)** Relative expression of CORO2A in malignant and non-malignant breast tissues in four GEO datasets, GSE21422, GSE29431, GSE42568, and GSE61304. Data are mean ± SEM. Student's *t*-test (unpaired) was adopted to compare the statistical significance. ***P < 0.001. Panels **(B–H)** show CORO2A transcript in subgroups of patients with breast cancer, stratified based on age of diagnosis, gender, and other criteria (UALCAN). **(B)** Relative expression of CORO2A in normal and breast cancer samples. **(C)** Relative expression of CORO2A in normal individuals or in breast cancer patients with different age. **(D)** Relative expression of CORO2A in normal individuals or in breast cancer patients with differential ethnicity. **(E)** Relative expression of CORO2A in normal individuals or in breast cancer patients with differential tumors stages. **(F)** Relative expression of CORO2A in normal individuals or in breast cancer patients with differential nodal metastatic status. **(G)** Relative expression of CORO2A in normal individuals or in breast cancer patients with differential tumors subclasses. **(H)** Relative expression of CORO2A in normal individuals or breast cancer patients with differential menopause status. Data are mean ± SEM. **P < 0.01; ***P < 0.001. **(I)** Comparison of CORO2A mRNA expression between breast cancer tissues and paracancerous tissues (*n* = 22). β-actin was used as an internal reference gene for normalization. Data were analyzed using paired Student's *t*-test.

**Figure 2:**
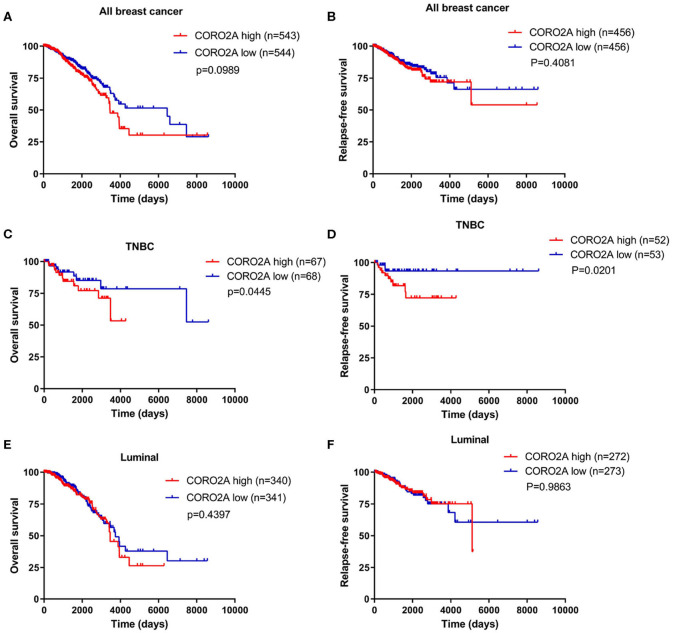
Kaplan–Meier survival analysis of breast cancer patients based on CORO2A expression level. **(A)** Comparison of overall survival (OS) between patients with CORO2A high and low expression levels in 1,087 breast cancer patients. **(B)** Comparison of relapse free survival (RFS) between patients with CORO2A high and low expression levels in 912 breast cancer patients. **(C)** Comparison of overall survival (OS) between patients with CORO2A high and low expression levels in 135 TNBC patients. **(D)** Comparison of relapse free survival (RFS) between patients with CORO2A high and low expression levels in 105 TNBC patients. **(E)** Comparison of overall survival (OS) between patients with CORO2A high and low expression levels in 681 luminal breast cancer patients. **(F)** Comparison of relapse free survival (RFS) between patients with CORO2A high and low expression levels in 545 luminal breast cancer patients. The samples with absent relapse free survival (RFS) time are removed in advance, when comparing the relapse free survival (RFS) between patients with CORO2A high and low expression levels.

In a previous study, we determined that the CORO2A transcription level was consistently upregulated in multiple breast cancer cohorts from the four Gene Expression Omnibus (GEO) and TCGA databases (4). These four datasets were generated from the same detecting microarray platform: [HG-U133_Plus_2] Affymetrix Human Genome U133 Plus 2.0 Array. Therefore, there is a smaller difference between batches. The expression level of CORO2A in those Gene Expression Omnibus (GEO) datasets was visualized and shown in Figure 1A in this study. Moreover, in the current study, further subgroup analysis of multiple clinicopathological features of breast cancer samples in TCGA consistently showed elevated level of CORO2A transcript. The level of CORO2A transcript was significantly upregulated in breast cancer tissue compared with normal tissue in subgroup analyses based on age, ethnicity, tumor stage, nodal metastatic status, disease subclass, and menopause status (Figures 1B–H). Significant overexpression of CORO2A was also validated in breast cancer samples (*n* = 22) collected in our clinic (Figure 1I). Moreover, the prognostic value of CORO2A was explored by the analysis of TCGA database. Although no significant association was observed between CORO2A mRNA expression and overall survival (OS) or relapse-free survival (RFS) in breast cancer patients (Figures 2A,B). However, Kaplan–Meier survival curves of CORO2A in breast cancer patients with differential tumor subclasses revealed that elevated expression of CORO2A indicated shorter OS in triple-negative breast cancer (TNBC) patients (Figure 2C). Moreover, the median RFS time of TNBC patients with high CORO2A mRNA expression was significantly shorter than those with low expression (Figure 2D). Unfortunately, these findings were not observed in luminal breast cancer patients (Figures 2E,F). To rule out the potential influence of age, pathologic stage, and lymphnodes states on prognosis of patients, Cox proportional hazards regression was further performed in TNBC. The results showed that CORO2A level was independent predictor for OS (HR = 2.151, 95% CI: 1.096–6.052, *p* = 0.037) and RFS (HR = 4.607, 95% CI: 1.214–17.486, *p* = 0.025) in TNBC patients (Tables 2, 3). Thus, these data suggest that high CORO2A expression may function as a potential diagnostic indicator in TNBC.

**Table 2:** Cox proportional hazards regression analysis for OS in TNBC patients.

**Variable**	**Univariate analysis**		**Multivariate analysis**	
	**HR (95% CI)**	***p*-value**	**HR (95% CI)**	***p*-value**
CORO2A expression	1.730 (1.067–4.411)	0.049	2.151 (1.096–6.052)	0.037
Age	1.154 (0.442–3.011)	0.770	1.091 (0.372–3.204)	0.874
Pathologic stage	3.996 (1.854–8.611)	<0.001	3.618 (1.285–10.189)	0.015
Lymphnodes states	4.274 (1.620–11.277)	0.003	1.920 (0.543–6.790)	0.311

**Table 3:** Cox proportional hazards regression analysis for RFS in TNBC patients.

**Variable**	**Univariate analysis**		**Multivariate analysis**	
	**HR (95% CI)**	***p*-value**	**HR (95% CI)**	***p*-value**
CORO2A expression	3.556 (1.076–12.955)	0.025	4.607 (1.214–17.486)	0.025
Age	0.846 (0.233–3.076)	0.800	0.851 (0.232–3.127)	0.808
Pathologic stage	4.964 (1.863–13.227)	0.001	3.809 (1.036–14.010)	0.044
Lymphnodes states	3.571 (1.166–10.936)	0.026	2.090 (0.464–9.419)	0.337

### Analyses of the Intersection of CORO2A-coexpressed Genes and Differentially Expressed Genes After CORO2A Silencing in Human Breast Cancer

**Figure 3:**
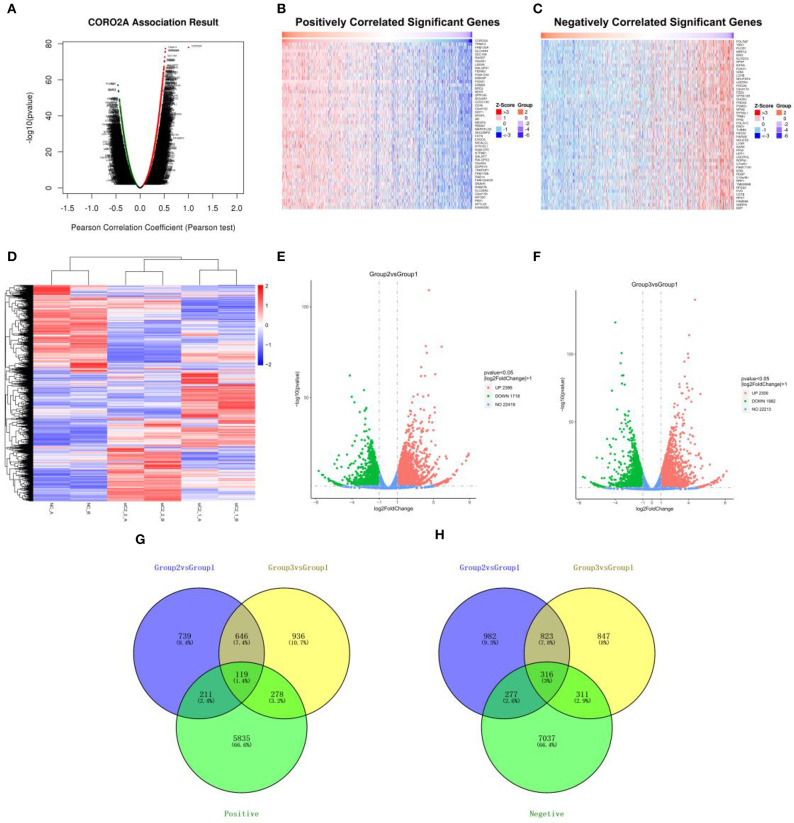
Analyses of the intersection of CORO2A-coexpressed genes and differentially expressed genes after CORO2A silencing in human breast cancer. **(A)** CORO2A-coexpressed genes in breast cancer were analyzed by Pearson test (LinkedOmics). **(B)** TOP 50 genes positively correlated with CORO2A expression in breast cancer (LinkedOmics). **(C)** TOP 50 genes negatively correlated with CORO2A expression in breast cancer (LinkedOmics). Red represents genes positively correlated with CORO2A expression, while green represents genes negatively correlated with CORO2A expression. **(D)** Shows a heat map from hierarchical clustering of differentially expressed genes in CORO2A-knockdown BT549 cells vs. BT549 control cells (RNA-seq). Red color represents upregulated genes and blue color represents downregulated genes. Each row represents a single gene. **(E,F)** show the Volcano plots of differentially expressed genes after CORO2A silencing (RNA-seq). Red color represents upregulated genes and blue color represents downregulated genes. **(E)** Volcano plots of Group2 vs. Group1; **(F)** Volcano plots of Group3 vs. Group1. Panels **(G,H)** are Venn diagram depicting the intersecting genes. **(G)** Venn diagrams of intersecting genes of downregulated genes after CORO2A silencing and genes positively correlated with CORO2A expression; **(H)** Venn diagrams of intersecting genes of upregulated genes after CORO2A silencing and genes negatively correlated with CORO2A expression. The blue circle and the yellow circle represent the differentially expressed genes between CORO2A-knockdown BT549 cells vs. BT549 control cells, and the green circle represents CORO2A-coexpressed genes. siC2_1_A or siC2_1_B: transfected with CORO2A siRNA-1 (si-CORO2A-1); siC2_2_A or siC2_2_B: transfected with CORO2A siRNA-2 (si-CORO2A-2); NC_A or NC_B: transfected with unspecific siRNA; Group2 vs. Group1: comparison of group transfected by si-CORO2A-1 and group transfected by si-NC; Group3 vs. Group1: comparison of group transfected by si-CORO2A-2 and group transfected by si-NC; Positive: genes positively correlated with CORO2A expression; Negative: genes negatively correlated with CORO2A expression.

The *Function* module of LinkedOmics was applied to analyze mRNA sequencing data from 1,093 breast cancer patients in the TCGA. As shown in the volcano plot (Figure 3A), 5,596 genes (dark red dots) showed significantly positive correlations with CORO2A, whereas 6,835 genes (dark green dots) showed significantly negative correlations (false discovery rate [FDR] < 0.01). The top 50 genes significant positively and negatively correlated with CORO2A are shown in the heat map (Figures 3B,C). This result indicates a widespread impact of CORO2A on the transcriptome. The statistical scatter plots for individual genes of TRIM14, FAM120A, SLC44A4, and SEC16A are shown in Supplementary Figures 1A–D. CORO2A expression showed a strong positive correlation with the expression of TRIM14 (positive rank #1, Pearson correlation = 0.523, *p* = 5.23E−78), FAM120A (Pearson correlation = 0.519, *p* = 2.39E−76), SLC44A4 (Pearson correlation = 0.518, *p* = 4.04E−76), and SEC16A (Pearson correlation = 0.510, *p* = 2.62E−73).

To further gain insight into the functional roles of CORO2A, RNA sequencing analysis in CORO2A-knockdown BT549 cells vs. BT549 control cells was performed. We identified 765 genes that were significantly downregulated and 1,139 genes that were significantly upregulated (*P* < 0.05; fold change > 2) between BT549/si-CORO2A and BT549/NC cells (Figures 3D–H). After overlapping genes coexpressed with CORO2A, a total of 119 genes were identified to be downregulated after CORO2A silencing and positively correlated with CORO2A expression, while a total of 316 genes were identified to be upregulated after CORO2A silencing and negatively correlated with CORO2A expression (Figures 3G,H). This result indicated that these 435 differentially expressed genes (DEGs) were extremely likely to be involved in the functional networks of CORO2A.

### Enrichment Analysis of CORO2A Functional Networks in Breast Cancer

**Figure 4:**
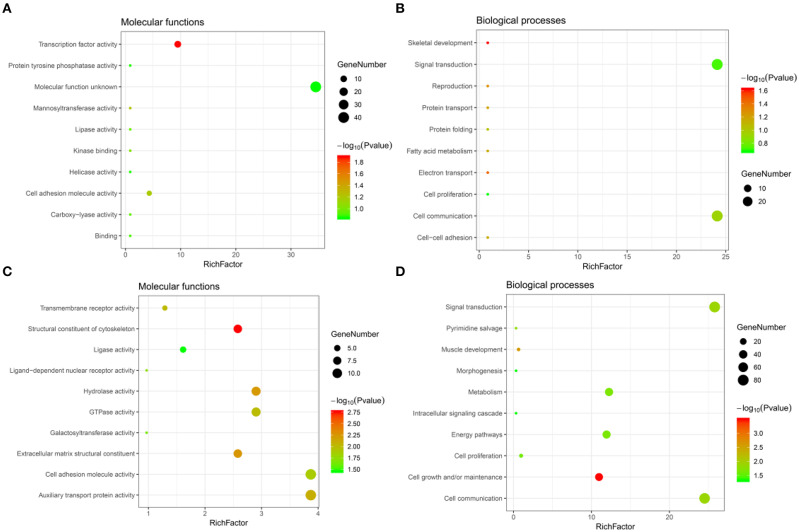
GO annotations analysis of the intersecting genes. **(A,B)** Top 10 elements significantly enriched in the GO categories by intersecting genes of downregulated genes after CORO2A silencing and genes positively correlated with CORO2A expression: **(A)** molecular functions, **(B)** biological processes. **(C,D)** Top 10 elements significantly enriched in the GO categories by intersecting genes of upregulated genes after CORO2A silencing and genes negatively correlated with CORO2A expression: **(C)** molecular functions, **(D)** biological processes. Significantly enriched elements are indicated in *Y*-axis. Rich factor in the *X*-axis represents the enrichment levels. The larger value of Rich factor represents the higher level of enrichment. The color of the dot stands for the different *P*-value and the size of the dot reflects the number of target genes enriched.

Significant GO term analysis by *FunRich* showed that genes downregulated after CORO2A silencing and positively correlated with CORO2A expression participated primarily in skeletal development, electron transport, reproduction, protein transport, and cell-cell adhesion. They act as transcription factors (Figures 4A,B and Supplementary Table 3). Genes upregulated after CORO2A silencing and negatively correlated with CORO2A expression primarily participated in cell growth and/or maintenance, muscle development, signal transduction, and cell communication. They act as structural constituents of the cytoskeleton and extracellular matrix structural constituents (Figures 4C,D and Supplementary Table 4).

**Figure 5:**
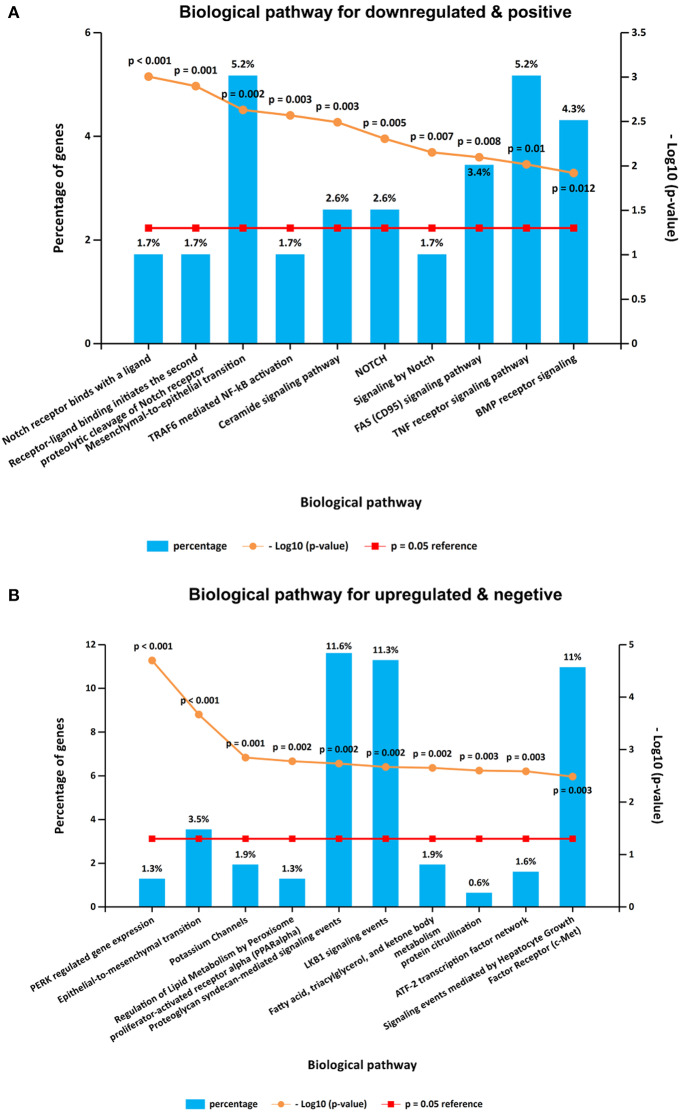
KEGG pathway enrichment analysis of the intersecting genes. **(A)** Top 10 elements significantly enriched in the KEGG categories by intersecting genes of downregulated genes after CORO2A silencing and genes positively correlated with CORO2A expression with a *p* < 0.05. **(B)** Top 10 elements significantly enriched in the KEGG categories by intersecting genes of upregulated genes after CORO2A silencing and genes negatively correlated with CORO2A expression with a *p* < 0.05.

It is interesting that KEGG pathway analysis of genes that were downregulated after CORO2A silencing and positively correlated with CORO2A expression showed significant enrichment in the following terms: notch receptor binds with a ligand, receptor-ligand binding initiates the second proteolytic cleavage of Notch receptor, and mesenchymal-to-epithelial transition pathways (Figure 5A and Supplementary Table 3); KEGG pathway analysis of genes that were upregulated after CORO2A silencing and negatively correlated with CORO2A expression showed significant enrichment in PERK-regulated gene expression, epithelial-to-mesenchymal transition, and potassium channel pathways (Figure 5B and Supplementary Table 4). This result indicated that CORO2A was extremely likely to be involved in the epithelial-to-mesenchymal transition process and might have a significant effect on the migration of breast cancer cells.

### The Effect of CORO2A on Cell Migration in Breast Cancer Cells

**Figure 6:**
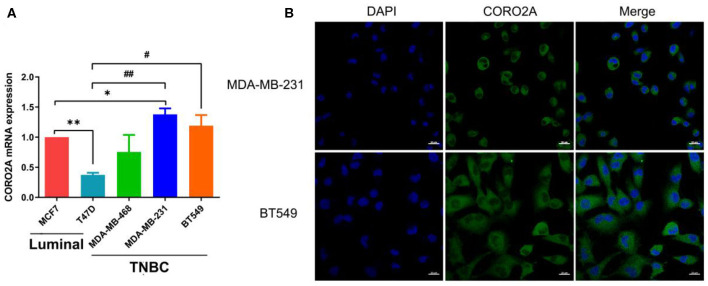
Expression and subcellular localization of CORO2A in breast cancer cells. **(A)** Relative expression of CORO2A was detected by using qRT-PCR in the luminal (MCF7 and T47D) and TNBC (MDA-MB-468, MDA-MB-231, and BT549) cell lines. Data are mean ± SEM and analyzed using one-way ANOVA. *P < 0.05; **P < 0.01 compared to MCF7; ^#^*P* < 0.05; ^##^*P* < 0.01 compared to T47D. **(B)** Subcellular localization of CORO2A was detected in MDA-MB-231 and BT549 cells by immunofluorescence staining. Scale bar, 20 μm.

**Figure 7:**
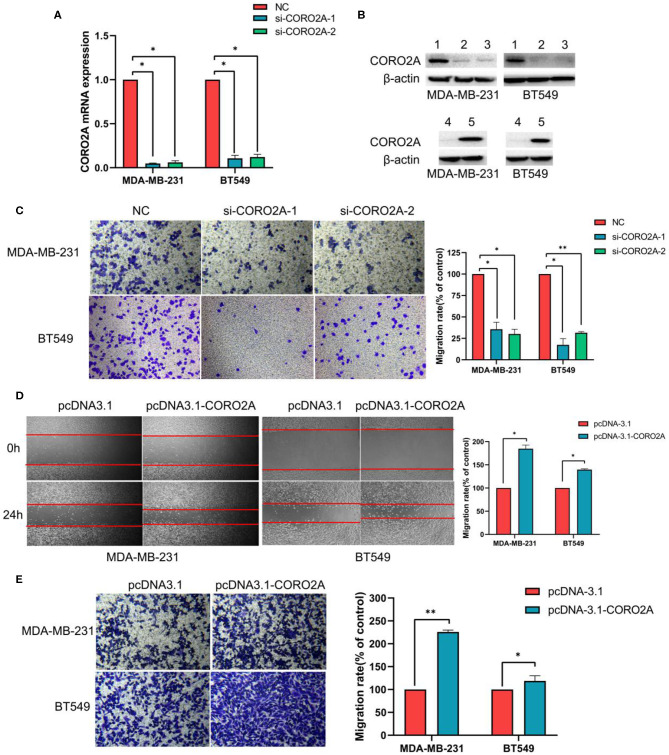
Effect of CORO2A on cell migration in TNBC cell lines. **(A)** After transfection with CORO2A siRNA for 72 h, the mRNA expression level of CORO2A was assessed by RT-qPCR in MDA-MB-231 and BT549 cells. **(B)** After transfection with CORO2A siRNA or plasmid for 72 h, the protein expression level of CORO2A was assessed by Western blotting in MDA-MB-231 and BT549 cells. Lanes in Western blotting are denoted as: lane 1—NC, lane 2—si-CORO2A-1, lane 3—si-CORO2A-2, lane 4—pcDNA-3.1, and lanes 5—pcDNA-3.1-CORO2A. **(C)** Representative images of cell migration assays and the mean ± SEM (*n* = 3) of three independent experiments of quantitative cell migration assays in MDA-MB-231 and BT549 cells are shown following CORO2A knockdown. Cell migration measured by scratch-wound **(D)** and Transwell asssay **(E)** are shown following CORO2A overexpression in MDA-MB-231 and BT549 cells. *P < 0.05, and **P < 0.01 compared to corresponding negative controls (NC) or pcDNA-3.1.

As previously mentioned, we have revealed that CORO2A is probably involved in the epithelial-to-mesenchymal transition process and might have a significant effect on the cell migration of breast cancer (Figures 5A,B and Supplementary Tables 3, 4). Besides, CORO2A was upregulated and that the elevated expression of CORO2A was associated with poor overall survival (OS) and relapse-free survival (RFS) in TNBC patients. And we assessed the expression and subcellular localization of CORO2A in breast cancer cells and found that the CORO2A had a relative higher expression in TNBC than in luminal breast cancer cell lines (Figure 6A). Immunofluorescence assay showed that CORO2A was mainly accumulated in the cytoplasm of breast cancer cells (Figure 6B). TNBC is associated with poor prognosis and belongs to aggressive tumor subtype, due to aggressive tumor phenotype(s), early metastasis to brain or visceral organ, and lack of clinically established targeted therapies (16, 17). Therefore, we first assessed the effect of CORO2A on cell migration in TNBC cell lines. Transwell assays were performed in MDA-MB-231 and BT549 TNBC cells. The knockdown of CORO2A at the mRNA and protein levels (Figures 7A,B) produced a decrease in migration ability (Figure 7C) in both breast cancer cell lines. In contrast, overexpression of CORO2A at the protein level (Figure 7B) produced an opposite effect on cell migration in these breast cancer cells (Figures 7D,E). These results indicate that CORO2A could promote the migration of breast cancer cells.

### The Effect of CORO2A on Cell Growth in Breast Cancer Cells

**Figure 8:**
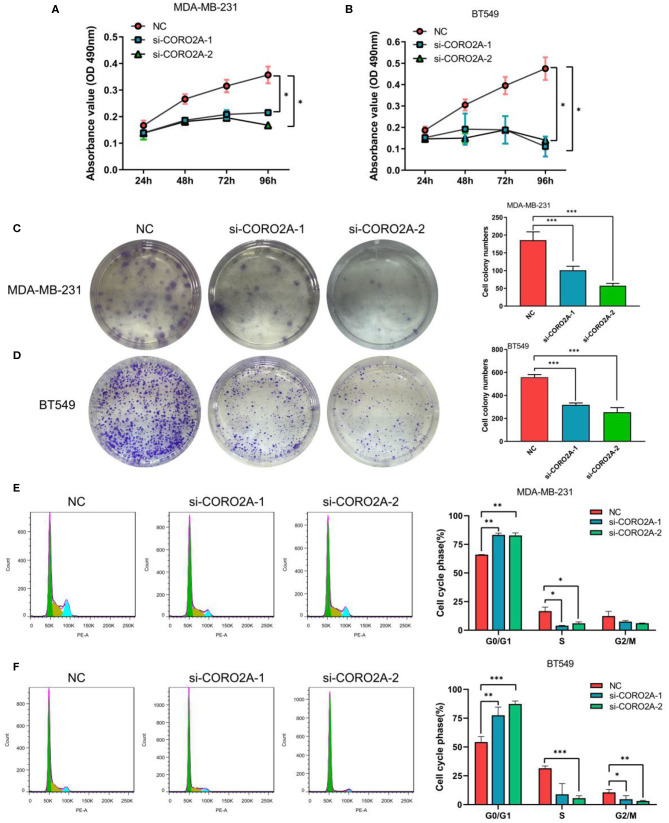
Effect of CORO2A knockdown on cell growth was evaluated by MTS, colony formation, and cell cycle distribution assays in TNBC cell lines. **(A,B)** CORO2A knockdown inhibited the cell viability of MDA-MB-231 and BT549 cells. Cell viability was measured using MTS assay and the data are mean ± SEM (*n* = 3) of three independent experiments. **(C,D)** Representative images of colony formation assay and the mean ± SEM (*n* = 3) of three independent experiments of quantitative colony formation assays in MDA-MB-231 **(C)** and BT549 **(D)** cells are shown following CORO2A knockdown. **(E,F)** Representative images of cell cycle distribution and the mean ± SEM (*n* = 3) of three independent experiments of quantitative cell cycle distribution in MDA-MB-231 **(E)** and BT549 **(F)** cells are shown following CORO2A knockdown. *P < 0.05, **P < 0.01, and ***P < 0.001 compared to corresponding negative controls (NC).

**Figure 9:**
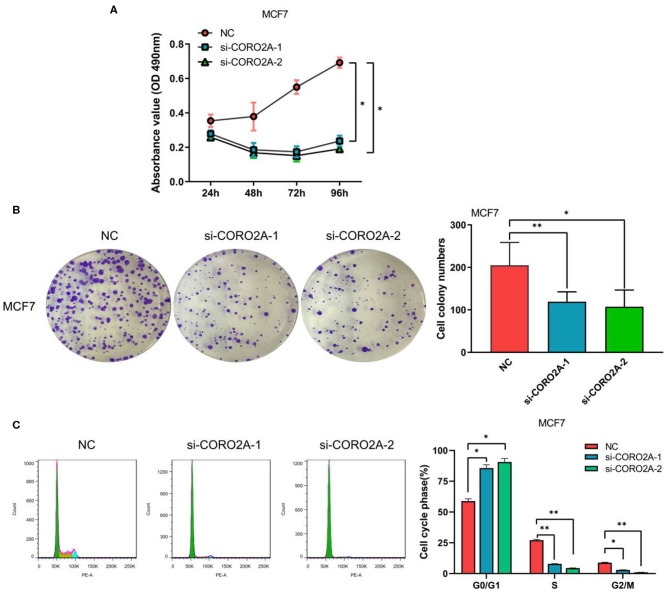
Effect of CORO2A knockdown on cell growth was evaluated by MTS, colony formation, and cell cycle distribution assays in luminal MCF7 breast cancer cells. **(A)** CORO2A knockdown inhibited the cell viability of MCF7 cells. **(B)** Representative images of colony formation assay and the mean ± SEM (*n* = 3) of three independent experiments of quantitative colony formation assays in MCF7 cells are shown following CORO2A knockdown. **(C)** Representative images of cell cycle distribution and the mean ± SEM (*n* = 3) of three independent experiments of quantitative cell cycle distribution in MCF7 cells are shown following CORO2A knockdown. *P < 0.05 and **P < 0.01 compared to corresponding negative controls (NC).

Furthermore, we also determined the effect of CORO2A on cell growth in human breast cancer cells. MTS, colony formation, and cell cycle distribution assays were performed in MDA-MB-231 and BT549 breast cancer cells, respectively. The knockdown of CORO2A produced a decrease in viability (Figures 8A,B) and colony formation (Figures 8C,D) in both breast cancer cell lines. The cell cycle distribution analysis showed that the decreased level of CORO2A could noticeably induce cell cycle arrest in the G0/G1 phase (Figures 8E,F). MTS, colony formation, and cell cycle distribution assays (Figures 9A–C) were also performed in luminal MCF7 breast cancer cells, and the observed effects were the same as those in MDA-MB-231 and BT549 breast cancer cells. However, the overexpression of CORO2A did not produce a significantly increased effect on cell viability in MDA-MB-231 and BT549 breast cancer cells (these data were not showed). These results indicate that knockdown CORO2A could inhibit the growth of both TNBC and luminal breast cancer cells, suggesting that the decrease of CORO2A level may have “inhibitor” function in breast cancer progress.

### CORO2A Networks of Transcription Factors or miRNA Targets in Breast Cancer

**Figure 10:**
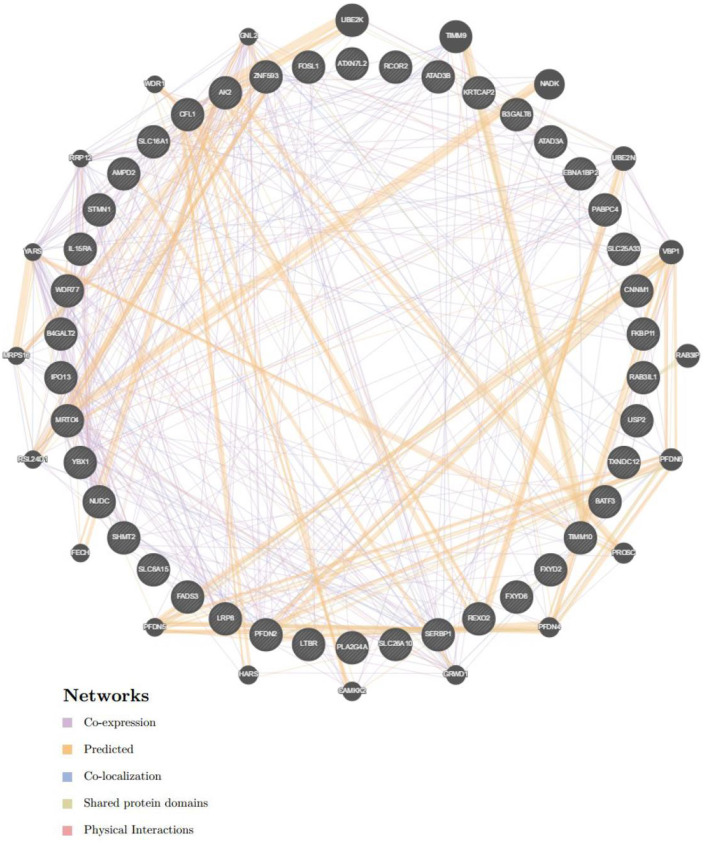
Protein-protein interaction network of transcription factor MYC-target networks construction (GeneMANIA). Protein-protein interaction (PPI) network illustrates the gene set that was enriched in the target network of MYC. Different colors of the network edge represent the bioinformatics methods applied including co-expression, website prediction, co-localization, shared protein domains, and physical interactions.

To further explore the targets of CORO2A in breast cancer, we analyzed the transcription factor and miRNA target networks of positively correlated gene sets generated by GSEA. The top four most significant transcription factor-target networks were related mainly to MYC, ROAZ, MYCMAX, and USF (Table 4 and Supplementary Tables 5, 6). The miRNA-target network was associated with (ATGTACA) miR-493, (TTGCACT) miR-130a, miR-301, miR-130b, and (TTTGCAC) miR-19a, miR-19b. In addition, the protein-protein interaction network constructed by GeneMANIA further showed a correlation among genes for the TF MYC_Q2 (Figure 10) and miRNA-493 (Supplementary Figure 2). Different colors of the network edge represent different bioinformatics methods applied, such as coexpression, website prediction, colocalization, shared protein domains, and physical interactions.

**Table 4:** The transcription factor- and miRNA-target networks of CORO2A in breast cancer (LinkedOmics).

**Enriched category**	**Geneset**	**LeadingEdgeNum**	**FDR**	***p*-value**
Transcription Factor Target	V$MYC_Q2	42	0.016	0
	V$ROAZ_01	5	0.017	0
	V$MYCMAX_01	71	0.031	0
	V$USF_C	76	0.032	0
miRNA Target	ATGTACA, MIR-493	115	0	0
	TTGCACT, MIR-130A, MIR-301, MIR-130B	132	0	0
	TTTGCAC, MIR-19A, MIR-19B	170	0	0
	GCACTTT, MIR-17-5P, MIR-20A, MIR-106A, MIR-106B, MIR-20B, MIR-519D	194	0	0
	TGCACTT, MIR-519C, MIR-519B, MIR-519A	145	0	0
	GTATGAT, MIR-154, MIR-487	30	0	0
	ACTGCAG, MIR-17-3P	35	0	0
	ACACTAC, MIR-142-3P	45	0	0

*LeadingEdgeNum represents the number of leading edge gene; FDR represents false discovery rate from Benjamini and Hochberg from gene set enrichment analysis (GSEA). V$ represents the annotation found in Molecular Signatures Database (MSigDB) for transcription factors (TF)*.

## Discussion

CORO2A, a novel component of the N-CoR (nuclear receptor co-repressor) complex, is associated with actin-dependent derepression of inflammatory response genes (6, 7). In the current study, we found that CORO2A was overexpressed in malignant breast tissues compared to normal breast tissues. Moreover, breast cancer patients with elevated expression of CORO2A had a poorer overall survival (OS) and relapse-free survival (RFS) in TNBC. Further bioinformatics analysis of public sequencing data and our own RNA-Seq data revealed that CORO2A is probably involved in the epithelial-to-mesenchymal transition process and might have a significant effect on the cell migration of breast cancer (Figures 5A,B and Supplementary Tables 3, 4). In addition, we also demonstrated *in vitro* that knockdown of CORO2A reduces cell migration and proliferation and induces cell cycle arrest in the G0/G1 phase in breast cancer cells (Figures 7–9). To gain more detailed insights into the potential functions of CORO2A in breast cancer and its regulatory network, bioinformatics analysis of public sequencing data was performed to guide future research in breast cancer.

Our study confirmed that the CORO2A mRNA level is elevated in breast cancer compared with normal breast tissue via analysis of transcriptional sequencing data from more than 1,000 clinical samples of breast cancer in the GEO and TCGA (Figure 1). The fold change of CORO2A was similar across the various breast cancer databases, and these results were also validated in the breast cancer samples (*n* = 22) collected in our clinic (Figure 1I). This finding suggests that the overexpression of CORO2A occurs in many cases of breast cancer and deserves further clinical support as a potential diagnostic and prognostic marker.

It is interesting that KEGG pathway analysis of genes that were downregulated after CORO2A silencing and positively correlated with CORO2A expression showed significant enrichment in the following terms: notch receptor binds with a ligand, receptor-ligand binding initiates the second proteolytic cleavage of the Notch receptor, and mesenchymal-to-epithelial transition pathways (Figure 5A and Supplementary Table 3); however, KEGG pathway analysis of genes that were upregulated after CORO2A silencing and negatively correlated with CORO2A expression showed significant enrichment in the PERK regulated gene expression, epithelial-to-mesenchymal transition, and potassium channel pathways (Figure 5B and Supplementary Table 4). This result indicated that CORO2A was probably involved in the epithelial-to-mesenchymal transition process and might have a significant effect on the migration of breast cancer cells. Thus, we validated the effects of CORO2A on cell migration and proliferation as well as on the cell cycle (Figures 7–9).

CORO2A participates in several important physiological functions, and its alteration may lead to changes of various downstream signaling pathways. GSEA enrichment analysis of target gene sets can help uncover important networks of transcription factors and miRNAs. Abnormal MYC expression is frequently involved in the initiation and development of breast cancer. MYC expression is elevated in TNBC and has been reported as one of the key features driving TNBC (18, 19). Oncogenic MYC directly transcriptionally activates the IRE1/XBP1 pathway by binding to the IRE1 promoter and enhancer (20). Wang et al. (21) revealed that overexpression of lncRNA EPIC1 is associated with poor prognosis in luminal B breast cancer patients and enhances tumor growth *in vitro* and *in vivo*. Mechanistically, EPIC1 promotes cell cycle progression by interacting with MYC, and EPIC1 knockdown reduces the occupancy of MYC on its target genes. Furthermore, Lee et al. (22) demonstrated that MYC and MCL1 confer resistance to chemotherapy by expanding CSCs via mitochondrial oxidative phosphorylation (mtOXPHOS) and that targeting mitochondrial respiration and HIF-1α may reverse chemotherapy resistance in TNBC. Therefore, our analyses suggest that MYC is an important target of CORO2A and that CORO2A acts through this factor to regulate the cell migration, cell cycle, and proliferation capacity of breast cancer. Further studies should evaluate this hypothesis.

Our study identified several miRNAs that were associated with CORO2A. MicroRNAs are mainly involved in the posttranscriptional regulation of gene expression and have potential applications for the clinical assessment of patient outcome in cancer, as well as in cancer monitoring and therapy (23). The particular microRNAs in our study were associated with tumor proliferation, migration, invasion, metastasis, chemoresistance, and angiogenesis (24–27). In fact, microRNA-493-5p was identified as most prominently upregulated in male breast cancer (28). MicroRNA-493 is a prognostic factor in TNBC and patients with high microRNA-493 expression have better disease-free survival than patients with low microRNA-493 expression (29). MicroRNA-493 and microRNA-19a can be used as diagnostic and prognostic markers of breast cancer (29, 30). MicroRNA-493-5p, microRNA-130a, microRNA-130b-3p, microRNA-301, and microRNA-19a-3p suppress the migration and invasion of breast cancer cells (31–35). Dysregulation of these miRNAs is consistent with the phenotype of CORO2A upregulation in breast cancer.

This study provides multilevel evidence for the importance of CORO2A in tumorigenesis and its potential as a marker in breast cancer. CORO2A is specifically related to several tumor-associated miRNAs (such as miRNA-493) and transcription factors (such as MYC). This study uses online tools and RNA-seq assays to perform target gene analyses on tumor data. Compared with traditional chip screening, this method has the advantages of large sample size, low cost, and simplicity. This enables large-scale genomics research as well as subsequent mechanistic studies of CORO2A in breast cancer. Nevertheless, whether breast cancer patients overexpressing CORO2A could benefit from the suppression of CORO2A or whether CORO2A is a promising therapeutic target still needs further experimental support, including preclinical and prospective clinical studies.

## Data Availability Statement

Publicly available datasets were analyzed in this study. This data can be found here: http://ualcan.path.uab.edu.; http://tcga-data.nci.nih.gov; http://www.linkedomics.org/login.php.

## Ethics Statement

The studies involving human participants were reviewed and approved by the Ethics Committee of Xiangya Hospital. The patients/participants provided their written informed consent to participate in this study.

## Author Contributions

J-LD conceived, designed the research, conducted the experiments, analyzed and interpreted data, and wrote the manuscript. H-BZ, YZ, Y-HX, and YH participated in analyzing and interpreting the data. GW participated in the experimental design and provided financial support. All authors read and approved the final manuscript.

## Conflict of Interest

The authors declare that the research was conducted in the absence of any commercial or financial relationships that could be construed as a potential conflict of interest.
