# Supplementary material for: Effects of CORO2A on Cell Migration and Proliferation and Its Potential Regulatory Network in Breast Cancer
Source: Front Oncol. 2020 Jun 26;10:916. doi: 10.3389/fonc.2020.00916 (PMC7333780; doi:10.3389/fonc.2020.00916)
Supplement: Supplementary file 1 [file Table_1.docx]

**Supplementary Table 1.** Clinicopathological characteristics of 22 pairs breast cancer patient.

| **Characteristics** | **Number of case (%)** | **Characteristics** | **Number of case (%)** |
| --- | --- | --- | --- |
| **ER** |  | **Pathologic_M** |  |
| negative | 5 (22.7) | M0 | 3 (13.6) |
| positive | 15 (68.1) | M1 | 2 (9.0) |
| unknown | 2 (9.0) | unknown | 17 (77.2) |
| **PR** |  | **Pathologic_T** |  |
| negative | 8 (36.4) | T1 | 0 (0) |
| positive | 11 (50.0) | T2 | 3 (13.6) |
| unknown | 3 (13.6) | T3 | 1 (4.5) |
| **HER2** |  | T4 | 1 (4.5) |
| negative | 1 (4.5) | unknown | 17 (77.2) |
| positive | 11 (50.0) | **Pathologic_stage** |  |
| unknown | 10 (45.4) | Stage1 | 0 (0) |
| **Ki67** |  | Stage2 | 19 (86.3) |
| <15% | 7 (31.8) | Stage3 | 0 (0) |
| 15-30% | 7 (31.8) | Stage4 | 0 (0) |
| >30% | 3 (13.6) | unknown | 3 (13.6) |
| unknown | 5 (22.7) | **Size(cm)** |  |
| **Age(y)** |  | <2 | 1（4.5） |
| <60 | 19 (86.3) | >=2 | 21（95.4） |
| >=60 | 3 (13.6) |  |  |
| **Pathologic_N** |  |  |  |
| N0 | 1 (4.5) |  |  |
| N1 | 4 (18.1) |  |  |
| N2 | 0 (0) |  |  |
| N3 | 0 (0) |  |  |
| unknown | 17 (77.2) |  |  |
