# Supplementary material for: Effects of CORO2A on Cell Migration and Proliferation and Its Potential Regulatory Network in Breast Cancer
Source: Front Oncol. 2020 Jun 26;10:916. doi: 10.3389/fonc.2020.00916 (PMC7333780; doi:10.3389/fonc.2020.00916)
Supplement: Supplementary file 2 [file Table_2.docx]

**Supplementary Table 2.** Primer sequences used for qRT-PCR.

| **Gene symbol** | **Primer sequence** |
| --- | --- |
| CORO2A | F: 5′-ATCCTCTTCAGTGCTGGCTAT-3′  R: 5′-TAACCCGAATCTTGCGGTCTT- 3′ |
| β-actin | F: 5′-TTGATTTTGGAGGGATCTCGCTC-3′  R: 5′-GAGTCAACGGATTTGGTCGTATTG- 3′ |
