# Supplementary material for: Effects of CORO2A on Cell Migration and Proliferation and Its Potential Regulatory Network in Breast Cancer
Source: Front Oncol. 2020 Jun 26;10:916. doi: 10.3389/fonc.2020.00916 (PMC7333780; doi:10.3389/fonc.2020.00916)
Supplement: Supplementary file 5 [file Table_5.docx]

**Supplementary Table 5.** Significantly enriched transcription factor-target networks of CORO2A in breast cancer (LinkedOmics).

| **Geneset** | **LeadingEdgeGene** |
| --- | --- |
| V$MYC_Q2 | AK2;AMPD2;ATAD3A;ATAD3B;ATXN7L2;B3GALT6;B4GALT2;BATF3;CFL1;CNNM1;EBNA1BP2;FADS3;FKBP11;FOSL1;FXYD2;FXYD6;IL15RA;IPO13;KRTCAP2;LRP8;LTBR;MRTO4;NUDC;PABPC4;PFDN2;PLA2G4A;RAB3IL1;RCOR2;REXO2;SERBP1;SHMT2;SLC16A1;SLC25A33;SLC26A10;SLC6A15;STMN1;TIMM10;TXNDC12;USP2;WDR77;YBX1;ZNF593 |
| V$ROAZ_01 | DUSP7; FBXO32; HEBP2; RCOR2; TIMM50 |
| V$MYCMAX_01 | AMPD2;ARMC6;ATAD3A;ATAD3B;BCL7C;CAMKV;CD3EAP;CGREF1;CNNM1;CSK;CYP2D6;DAZAP1;DAZL;DCAF13;EEF1B2;EFNB1;ESRRA;EWSR1;FAM19A4;FKBP11;GCSH;GPM6B;GPS1;HMGA1;HOXA3;HSPBAP1;IFRD2;IPO13;KCMF1;KCNN4;KRTCAP2;LRP8;LYAR;LZTS2;MANF;MON1A;MRPL40;NOP56;NOP58;NUDC;ODC1;OSR1;PA2G4;PABPC1;PDIA2;PFN1;PRDX4;PRMT1;PRR7;PTMA;PUS1;QTRT1;RAB3IL1;RANBP1;RCOR2;REXO2;RFX4;RPS19;RSPO2;SERBP1;SIGMAR1;SLC25A32;SLC6A15;STMN1;TCOF1;TFAP4;TIMM10;TIMM8A;TRMT2A;TUBA4A;YEATS2 |
| V$USF_C | ADCY3;AEN;ALDH3B1;AMPD2;ATXN7L2;B4GALT2;BATF2;BATF3;C21orf91;CAMKV;CNNM1;CYP2D6;DAZAP1;DAZL;EEF1E1;EFNB1;EIF5A;ELK1;ELOVL4;ENPP6;EXOSC5;FADS3;FBL;FGF11;FKBP11;GABARAP;GCSH;GPX1;GRK6;HOXA3;HOXA4;HSPBAP1;HSPE1;IPO13;LPCAT4;LRP8;LTBR;LYAR;NUDC;OSR1;PA2G4;PABPC1;PABPC4;PAX6;PFDN2;PFDN6;PFN1;PLA2G4A;POLR2H;PRMT1;PSMB3;PTMA;RPL13A;RPL22;RPS19;RSPO2;RXRB;SEMA7A;SERBP1;SH3KBP1;SIGMAR1;SLC25A33;SLC39A7;SLC6A15;STMN1;TFAP4;TIMM10;TMEM132E;TNFRSF21;TXNDC12;UBXN1;USP2;VGF;WBP2;WDR46;YBX1 |
