# Supplementary material for: Effects of CORO2A on Cell Migration and Proliferation and Its Potential Regulatory Network in Breast Cancer
Source: Front Oncol. 2020 Jun 26;10:916. doi: 10.3389/fonc.2020.00916 (PMC7333780; doi:10.3389/fonc.2020.00916)
Supplement: Supplementary file 6 [file Table_6.docx]

**Supplementary Table 6.** Significantly enriched miRNA-target networks of CORO2A in breast cancer (LinkedOmics).

| **Geneset** | **LeadingEdgeGene** |
| --- | --- |
| ATGTACA,MIR-493 | ACAP2;ADAM10;ADCY1;AFF3;ANKRD17;ANKRD28;ANKRD50;AR;ARFGEF1;ARHGAP5;ARID1A;ARID1B;BAZ2A;BCL2;BCOR;BTAF1;C16orf45;CASC4;CCNT2;CDC73;CITED2;CNOT6;COBLL1;CREBBP;CSNK1A1;CSNK1A1L;CTDSPL;CTNND1;CTNND2;DACH1;DDX3X;DKK2;DNAJC13;DOCK9;DUSP16;ESRRG;FAM120A;FNIP1;FZD4;GAB1;GBF1;GPC4;HIPK1;KCNMA1;KDM5B;KDM6A;KIAA2026;KLF3;LARP4;LCA5;LRIG1;MADD;MAML3;MBD5;MED13;MED13L;MFSD6;NBEA;NCOA1;NDST1;NRXN3;NUDT4;NXPH1;OSBPL6;OVOL1;PCGF2;PDPK1;PDS5A;PHF12;PHF2;PIKFYVE;PPP3CB;PPTC7;PREX1;PUM2;RALGPS1;RBBP6;RBM12;REEP1;RHOT1;RNF38;RPS6KA5;RYBP;SEL1L;SIN3A;SIPA1L3;SLC22A23;SMG1;SNX9;SP3;SPEN;SPRED2;SPRYD3;SRPK2;SSH2;TAB3;TAOK3;TBK1;TJP1;TM9SF3;TMEM106B;TRPS1;UBE2Q2;UBE3A;UNC5A;USP32;VPS13D;WDFY3;ZBTB11;ZDHHC17;ZFC3H1;ZFX;ZMIZ1;ZNF385B;ZNF800 |
| TTGCACT,MIR-130A,MIR-301,MIR-130B | ABCC5;ABHD3;ACBD5;ACVR1;ADCY1;ANKRD12;ANKRD28;APPL1;ARFIP1;ARHGAP1;ARHGAP12;ARHGEF12;ARID4B;ASXL2;ATG16L1;ATRN;ATRX;ATXN1;BAHD1;BAZ2A;BIRC6;BMPR2;BPTF;BRWD1;BTAF1;BTBD7;C5orf30;CCDC126;CEP120;CHD9;CLIP1;CLOCK;CLTC;CNOT4;CNOT6;CPEB4;CSNK1G1;CUL3;DICER1;DIP2A;DNAJC16;DYNLL2;ELK3;ENPP5;EPC2;ERBB4;ESR1;EXOC5;FAM20B;FBXO28;G3BP2;GPATCH8;GPCPD1;HBP1;HOXB3;IKZF4;INO80;ITPK1;ITPR1;KLHL20;LNPEP;LRIG1;MAML1;MAP3K12;MAP3K9;MECP2;MFSD6;MIB1;MIER3;MLEC;MPHOSPH9;MTF1;MYB;MYT1;NAA30;NBEA;NCOA1;NIPA1;NPNT;NPTN;PAN3;PFKFB3;PHF12;PHF3;PIK3IP1;PTPRG;RAB30;RAB5B;RAP2C;RAPGEF4;RHOT1;RNF38;RXRA;SASH1;SFMBT1;SH3D19;SHANK2;SKP1;SLC44A1;SLMAP;SMAD5;SNPH;SNX27;SOCS6;SPATA2;SPEN;SPOPL;SPTY2D1;STARD13;SYBU;TBC1D8;TESK2;TNRC6A;TP53INP1;TRIM3;TRPS1;TSC1;USP32;USP33;VPS37A;WDFY3;WDR20;WDR47;WEE1;ZBTB4;ZFC3H1;ZFP91;ZFPM2;ZFYVE26;ZNF217;ZNF609;ZNF800 |
| TTTGCAC,MIR-19A,MIR-19B | ACBD5;ADCY1;ADCY9;ADIPOR2;ADNP;ADSS;AFF1;ANKRD12;ANXA7;APPL1;ARFGEF1;ARFIP1;ARHGAP1;ARHGAP12;ARHGEF12;ARID4B;ARPP19;ASXL2;ATG16L1;ATRX;ATXN1;BMPR2;BPTF;BRWD1;BTAF1;BTBD7;C5orf30;CAB39;CAMSAP1;CAST;CCDC126;CCNT2;CDK13;CEP350;CGN;CLIP1;CLOCK;CLTC;CNOT6;CPEB4;CREBL2;CS;CSNK1G1;DDX3X;DHX40;DICER1;DNAJC16;EDARADD;ELK3;ELL2;ELMOD2;ELOVL5;ENPP5;EPC2;EPN2;ERBB4;ESR1;EXOC5;FAM114A1;FBXO28;FBXO8;FEM1C;FNDC3A;FOXP1;G3BP2;GPCPD1;GPR137B;HBP1;HIPK1;HNRNPF;IGSF3;INO80;ITPR1;KIAA1217;KIF3A;KLHL20;LNPEP;LPP;LRIG1;MACF1;MAP3K12;MBD6;MECP2;MED13L;MEF2D;MFSD6;MIB1;MIER3;MLEC;MON2;MPHOSPH9;MYLIP;NAV3;NBEA;NIPA1;NPTN;NR4A2;NRK;OCRL;OLFM1;PCDHA6;PFKFB3;PHF12;PHF20;PIK3R3;PKNOX1;PLXNC1;PPP2R5E;PPTC7;PRICKLE2;PRRT3;PTPRG;RAB18;RAB2B;RAB33B;RAB5B;RALGPS1;RAP2C;RAPGEF4;RHOB;RIMKLA;RIN2;RNF111;RNF2;RNF38;RXRA;SBF2;SFMBT1;SH3D19;SHANK2;SLC9A1;SLMAP;SMAD5;SMARCA2;SNPH;SNX27;SPATA2;SPEN;SPRYD3;SUZ12;SYBU;SYT1;TBC1D8;TBK1;TESK2;TGOLN2;TNRC6A;TOR1B;TP53INP1;TRAK2;TRPS1;TSC1;UBL3;USP32;USP33;VPS4B;WDFY3;WDR20;WDR26;WDR44;WDR47;WEE1;ZBTB4;ZFP91;ZFYVE26;ZMYND11;ZNF217;ZNF518A;ZNF609;ZNF800 |
| GCACTTT,MIR-17-5P,MIR-20A,MIR-106A,MIR-106B,MIR-20B,MIR-519D | ABHD2;AFF4;ANKFY1;ANKRD28;ANKRD50;ANO6;APBB2;ARHGAP1;ARHGAP12;ARHGEF3;ARID4A;ARID4B;ATG16L1;ATP1A2;ATXN1;BAHD1;BCL2L11;BCL2L2;BMPR2;BTBD10;BTBD7;C16orf72;C6orf120;CADM2;CAMK2N1;CCND1;CCNG2;CCNT2;CELSR2;CEP120;CEP97;CHD9;CLOCK;CNOT4;CNOT6;CRIM1;CRY2;CSNK1G1;CTDSPL2;DAZAP2;DCAF8;DCUN1D3;DIP2A;DNAJC16;DNAJC27;EIF4G2;ELK3;ENPP5;FAM102A;FAM126B;FBXL5;FBXO21;FBXW11;FCHO2;FEM1C;FGD5;FNBP1L;FNDC3A;FOXA1;FOXJ3;FRMD6;GAB1;GABPB2;GBF1;GOSR1;GRHL2;HBP1;IKZF4;INO80;ITCH;KCNMA1;KIAA1522;KIF3B;KLF9;KLHL20;LIMA1;LPGAT1;LRIG1;LYPD6;MAP3K12;MAP3K2;MAP3K9;MAP7;MAPK9;MARCH8;MECP2;MIDN;MKNK2;MTMR3;NAA30;NAPEPLD;NBEA;NCOA3;NEDD4L;NEK9;NIPA1;NR2C2;NR4A2;NTN4;OCRL;ORMDL3;P2RX4;PAFAH1B2;PAPOLA;PARD6B;PCDHA6;PKNOX1;PLAGL2;PLEKHA3;PLS1;PPP2R2A;PPP6C;PREX1;PRR15;PTEN;PTPN3;PURA;RAB11FIP1;RAB30;RAB5B;RABEP1;RABGAP1;RAP2C;RAPGEF4;RB1;RBL2;RPS6KA5;RSRC2;RUNX1;SAR1B;SASH1;SEMA4B;SENP1;SFMBT1;SGMS1;SIPA1L3;SLC1A2;SLC22A23;SLC40A1;SLC4A7;SMAD5;SOCS6;SORL1;SPOPL;SPTY2D1;SSH2;STAT3;STYX;SYBU;TAOK2;TBC1D8B;TBC1D9;TBX3;TGOLN2;TMCC1;TMEM127;TNKS2;TNRC6A;TOPORS;TP53INP1;TP53INP2;TRIM3;TRIM36;TRIP11;TRPS1;TSG101;TSPAN9;TWF1;TXNIP;UBE2Q2;UBE3C;UBFD1;USP3;USP32;USP46;VANGL1;WDFY3;WEE1;YPEL2;ZBTB4;ZBTB41;ZBTB6;ZBTB7A;ZFP91;ZFPM2;ZFYVE26;ZHX2;ZNF148;ZNF217;ZNF236;ZNF652;ZNF704;ZNF800;ZNFX1 |
| TGCACTT,MIR-519C,MIR-519B,MIR-519A | ABHD2;ABHD3;ACVR1;ADIPOR2;AFF4;ANKFY1;ANKRD12;ANKRD50;ARHGAP1;ARHGAP12;ARHGAP29;ARID4A;ARID4B;ASXL2;ATG16L1;ATRN;BAHD1;BCL2L2;BIRC6;BMPR2;BRWD1;BTBD10;BTBD7;CDK12;CELSR2;CEP120;CEP97;CHD9;CNOT4;CNOT6;CPEB2;CSNK1G1;CUL3;DAZAP2;DDX3X;DIP2A;DNAJC16;EIF4G2;ELK3;ENPP5;EPB41L4B;EPC2;ERBB4;FAM126B;FBXL5;FBXW11;FCHO2;FNBP1L;FOXA1;FOXJ3;FRMD6;GBF1;GOLGA1;GOSR1;HBP1;HIF1AN;HOXB3;IKZF2;IKZF4;ITCH;ITPR1;KLF9;KLHL20;LARP4;LPGAT1;LRIG1;MAML1;MAP3K12;MAP3K2;MAP3K9;MAP7;MECP2;MFSD6;MIB1;MIER3;MYB;MYT1;NAA30;NBEA;NF1;NFIA;NIPA1;PAPOLA;PARD6B;PCDH20;PCDHA6;PIGS;PKNOX1;PLEKHA3;PREX1;PRR15;PTPRT;RAB5B;RAP2C;RAPGEF4;RB1;RBBP6;RICTOR;SASH1;SEMA4B;SFMBT1;SGMS1;SHANK2;SLC1A2;SLC40A1;SLC4A7;SMAD5;SNRK;SOCS6;SORL1;SP3;SPOPL;SPTY2D1;SSH2;STAT3;STYX;SYBU;SYT1;TAOK3;TBC1D9;TBX3;TESK2;TMEM127;TNRC6A;TOPORS;TRIM3;TSG101;TSPAN9;UBE3A;USP3;USP32;VAMP4;VANGL1;WDFY3;WDR20;WEE1;ZBTB4;ZFC3H1;ZFP91;ZFPM2;ZHX2;ZNF217;ZNF236;ZNF704;ZNFX1 |
| GTATGAT,MIR-154,MIR-487 | ACACA;ANKS1B;ARID1A;ARID2;BMI1;C6orf120;CAPRIN1;GALC;HNRNPH2;KCNMA1;KDM6A;KIAA1217;NLK;NPTN;PIK3C2B;PPM1A;PUM2;RBBP6;RC3H1;RNF38;SEMA3C;SIRT1;SP1;SP3;SPRED2;STYX;TMEM184B;TOP1;YTHDC1;ZNF281 |
| ACTGCAG,MIR-17-3P | ACACA;ANKRD50;ARID2;BRWD3;BTF3;CDK17;CNOT4;CNOT6;COL12A1;CTR9;FNBP1L;FOXP1;FZD4;KCNMA1;MAML3;MIER3;NKAIN1;OLFM1;PARD3B;PPP2CA;RAB11A;RAB21;RAP2C;RHOT1;RNF111;SNX27;SPRED2;TMEM62;TRAPPC10;UHMK1;USP33;VEZF1;VEZT;VPS36;ZNF423 |
| ACACTAC,MIR-142-3P | ADCY9;AFF1;ANK3;APC;ARL15;ATG16L1;BAZ1A;BCLAF1;BRWD3;BTBD7;CCNT2;CPEB2;EDEM3;EHF;FBXO3;FNBP1L;FNDC3A;GNAQ;GTF2A1;HECTD1;ITGAV;KDM6A;LCOR;LRRC1;MBD6;MGAT4A;PRLR;PUM1;RERE;RICTOR;S1PR3;SGMS1;SMG1;SPIN1;STAU1;STRN3;SYPL1;TAOK1;TIPARP;TIRAP;TP53INP2;TRPS1;TWF1;ZBTB41;ZNF217 |
