# Supplementary material for: Effects of CORO2A on Cell Migration and Proliferation and Its Potential Regulatory Network in Breast Cancer
Source: Front Oncol. 2020 Jun 26;10:916. doi: 10.3389/fonc.2020.00916 (PMC7333780; doi:10.3389/fonc.2020.00916)
Supplement: Supplementary file 7 [file Data_Sheet_1.docx]

**Effects of CORO2A on cell migration and proliferation and its potential regulatory network in breast cancer**

Jun-Li Deng ^1,2,3,4^, Hai-Bo Zhang ^1,2,3,4^，Ying Zeng ^1,2,3,4^， Yun-Hua Xu ^1,2,3,4^，Ying Huang ^1,2,3,4^，Guo Wang ^1,2,3,4^*

^1^ Department of Clinical Pharmacology, Xiangya Hospital, Central South University, 87 Xiangya Road, Changsha 410008, P. R. China; Junli-Deng@csu.edu.cn (J.D.); [niaoluo@126.com](mailto:niaoluo@126.com) (H.Z.); zengying2007@163.com [(Y.Z.);](mailto:(Y.Z.);niaoluo@126.com) [xyh0707@csu.edu.cn](mailto:xyh0707@csu.edu.cn) (Y.X.); huangying14@csu.edu.cn（Y.H.);

^2^ Institute of Clinical Pharmacology, Central South University, Hunan Key Laboratory of Pharmacogenetics, 110 Xiangya Road, Changsha 410078, P. R. China;

^3^ Engineering Research Center of Applied Technology of Pharmacogenomics, Ministry of Education, 110 Xiangya Road, Changsha 410078, P. R. China;

^4^ National Clinical Research Center for Geriatric Disorders, 87 Xiangya Road, Changsha 410008, Hunan, P.R. China;

***** Correspondence:

Guo Wang

Department of Clinical Pharmacology, Xiangya Hospital, Central South University, Changsha 410008, China; Institute of Clinical Pharmacology, Central South University, Hunan Key Laboratory of Pharmacogenetics, Changsha 410078, China

Tel +86-0731-84805380

Fax +0086-731-82354476

Email: [207082@csu.edu.cn](mailto:207082@csu.edu.cn)

**
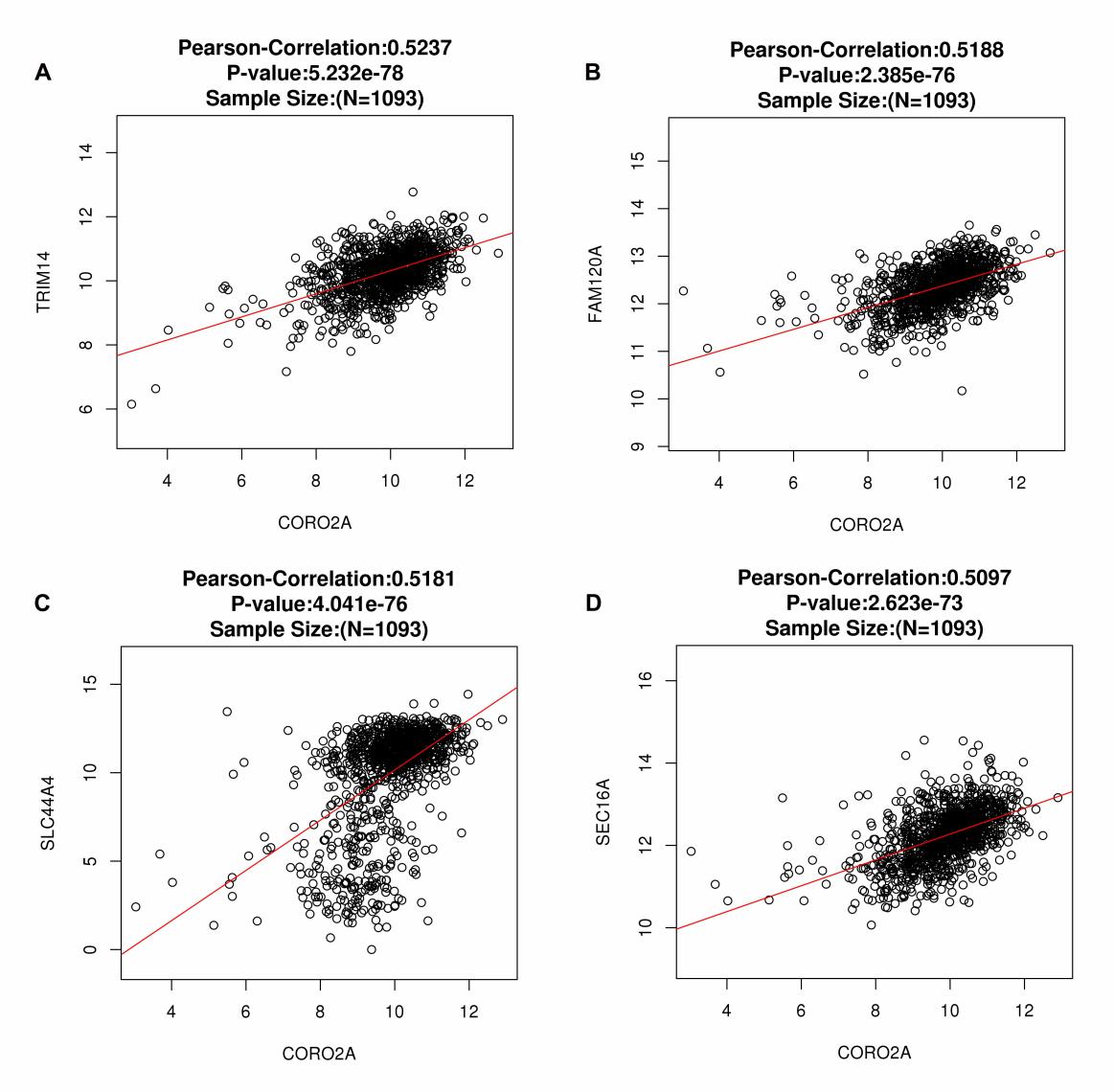
**

**Supplementary Figure 1. Gene expression correlation analysis for CORO2A, TRIM14, FAM120A, SLC44A4 and SEC16A (LinkedOmics).** The scatter plot shows Pearson correlation of CORO2A expression with expression of TRIM14 (A), FAM120A(B), SLC44A4 (C) and SEC16A (D).

**
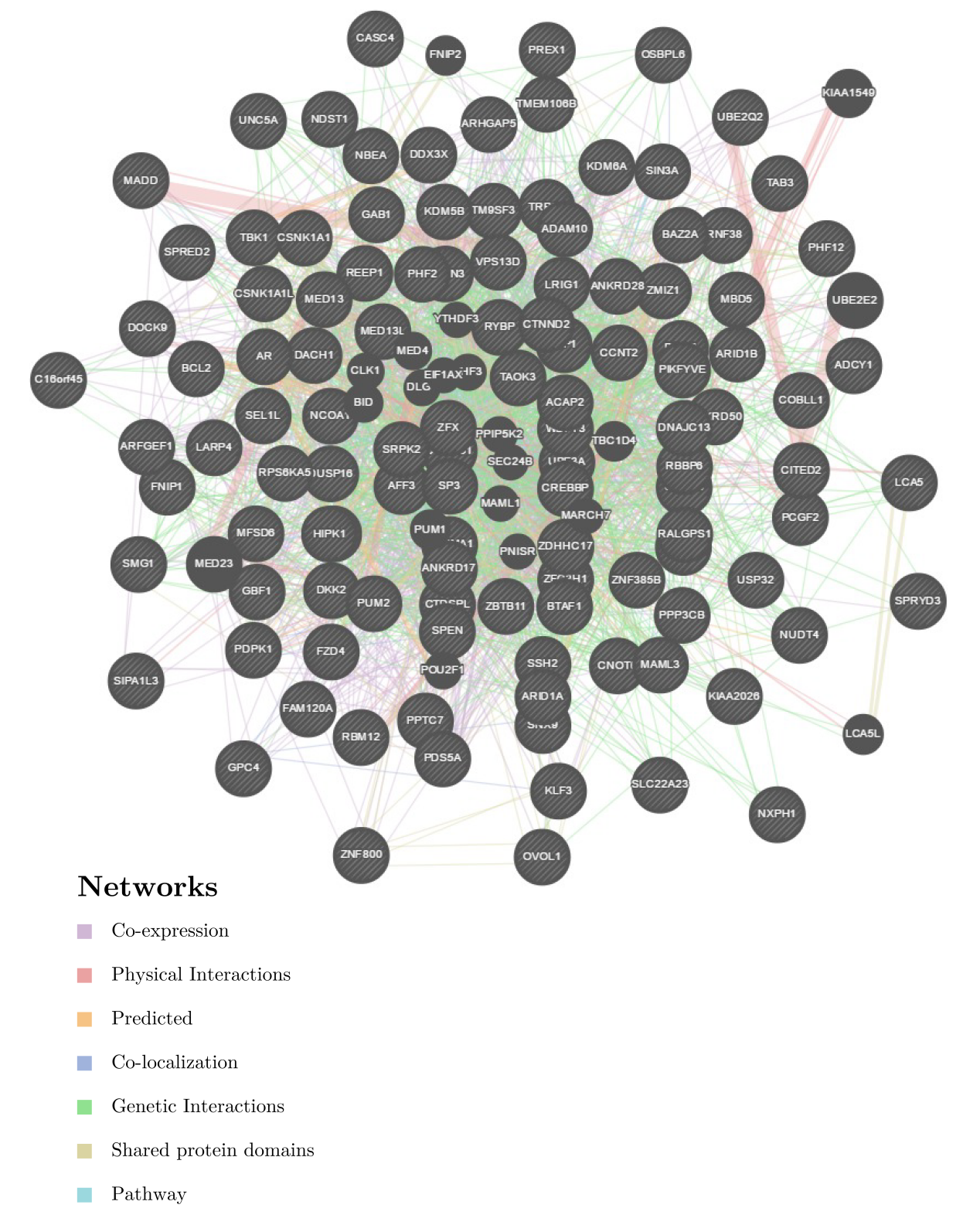
**

**Supplementary Figure 2.** **Protein-protein interaction network of miRNA-493-target networks construction (GeneMANIA).** Protein-protein interaction (PPI) network illustrates the gene set that was enriched in the target network of miRNA-493. Different colors of the network edge represent the bioinformatics methods applied including co-expression, physical interactions, predicted, co-localization, genetic interactions, shared protein domains and pathway.
